# Build Time Estimation for Fused Filament Fabrication via Average Printing Speed

**DOI:** 10.3390/ma12233982

**Published:** 2019-12-01

**Authors:** Gustavo Medina-Sanchez, Rubén Dorado-Vicente, Eloísa Torres-Jiménez, Rafael López-García

**Affiliations:** Department of Mechanical and Mining Engineering, University of Jaén, EPS de Jaén, Campus Las Lagunillas s/n, 23071 Jaén, Spain; gmedina@ujaen.es (G.M.-S.); etorres@ujaen.es (E.T.-J.); rlgarcia@ujaen.es (R.L.-G.)

**Keywords:** 3D printing, rapid prototyping, efficiency, printing time, experimental model

## Abstract

Build time is a key issue in additive manufacturing, but even nowadays, its accurate estimation is challenging. This work proposes a build time estimation method for fused filament fabrication (FFF) based on an average printing speed model. It captures the printer kinematics by fitting printing speed measurements for different interpolation segment lengths and changes of direction along the printing path. Unlike analytical approaches, printer users do not need to know the printer kinematics parameters such as maximum speed and acceleration or how the printer movement is programmed to obtain an accurate estimation. To build the proposed model, few measurements are needed. Two approaches are proposed: a fitting procedure via linear and power approximations, and a Coons patch. The procedure was applied to three desktop FFF printers, and different infill patterns and part shapes were tested. The proposed method provides a robust and accurate estimation with a maximum relative error below 8.5%.

## 1. Introduction

### 1.1. About Additive Manufacturing

Since the first 3D printer was developed in the early 80s, the number of additive manufacturing (AM) solutions, often called 3D printing methods in a non-technical context, and their applications do not stop increasing. It is noteworthy the potential of AM in different applications such as bio-printing [1,2], replicating broken objects or custom parts [3], experimental and educational demonstrators [4], rapid tooling [5] and so on.

According to the standard ISO/ASTM 52900-15 [6], AM solutions produce objects by joining materials, usually layer by layer, from 3D models. This standard classifies the existing solutions in seven types of processes considering how materials are deposited and bonded: material extrusion, binder jetting, material jetting, directed energy deposition, vat photopolymerization, powder bed fusion, and sheet lamination. The main AM advantages over traditional methods are low product development time, material savings, and capability to produce objects with complex shapes, enhanced density, and interior structures [7].

Fused filament fabrication (FFF) based on material extrusion processes is the most widespread AM technique [8], and low price models dominate the shipment numbers [9]. FFF consists of heating a thermoplastic filament, extruding the resulting melt and filling layer by layer a part following 2D paths while the plastic solidifies. Although less accurate than other AM technologies, FFF printers are broadly used because of their price [10], the wide range of plastics that can be printed, and the strength of the obtained parts [1,11].

### 1.2. Build Time

AM processes have several issues that limit their potential applications. AM issues in the spotlight are: development of new compatible materials [12], dimensional accuracy and surface roughness [13,14,15], mechanical properties of printed parts, discretization of CAD model and printed object, printer capabilities, maintenance, optimization of shape and part orientation, and time to manufacture a part (build time) [16,17].

Regarding build time, an accurate estimation can help firms to enhance their processes planning, and to compare AM solutions [18]. Moreover, these estimations can lead to more meaningful results in works dealing with the optimization of process parameters with the target to reduce the operation time [19], as well as those studies that consider the influence of printing speed and, therefore, time in the printed part appearance [20] or strength [21]. Finally, the costs of AM machine-hours depends on build time [22], and it stands to reason that, its contribution to the overall costs will increase the price of the used AM technology. Despite the previous arguments, build time has received less attention than other issues, such as dimensional accuracy and mechanical properties [23].

Time estimation is not a simple task because it depends on the printer and its control characteristics, as well as the printing parameters and the machine path planning. Moreover, time prediction is, in general, not accurate [24,25]. The simplest build time estimation is calculated as the total motion path length divided by the programmed printing speed and, in some cases, can differ more than 30% from the actual build time.

During the last decade, several researchers are concerned about build time estimation in additive manufacturing. According to the detailed work of Zhang et al. [26], there are three main strategies to determine the build time:

- Analytical-approaches: Define complex analytical models that describe in detail the printer kinematics and, therefore, allow build time estimation. These are the most accurate solutions, but the construction is complex (it depends on many data and on knowing the printing path in advance), it is applicable to a specific system and the prediction depends on the nominal values of the machine parameters and its control, which could differ from their actual values, increasing the estimation error.
- Parameter-approaches: Determine simple analytical relations between time and a selected set of factors that depends mainly on part geometry such as height, surface, and volume. Although its implementation is simple, the accuracy is low and, again, depends on each system.
- Experimental-approaches: Fit the real system response for different values of a set of parameters, usually shape factors such as in parameter-approaches.

Experimental solutions are more accurate than parameter ones and simpler than analytical ones, but there are no rules for data selection neither for the fitting strategy and, therefore, the repeatability is low. A change in the printing parameters forces to construct a new response function, therefore, experimental methods are not flexible.

The method developed by Zhang et al. [26], which is based on Grey theory, is an interesting improvement of the experimental approaches. The authors claim that their estimation error has an average value of 10% and it is better than other existing approaches. On the other hand, it is not well established how to select the input factors and many shape parameters are needed (part volume, support volume, part surface, part height, support height, and part projected area).

Later works to those reported in Zhang’s paper determine the build time according to the aforementioned strategies. Zhu et al. [27] developed both, an experimental and a parametric model. The last one is based on a reduced number of printing parameters (volume, height, and density) selected depending on their influence on the build time.

Different strategies are used to build experimental models. For example, the experimental solution explained by Zhu et al. [27] proposes a multi-factor regression, and Mohamed et al. [28] used a Q-optimal response surface methodology.

Examples of analytical methods are the estimations proposed by Habib and Khoda [29] or Komineas et al. [30]. The model developed by Komineas et al. [30] for material extrusion processes is based on a trapezoidal speed profile. It considers that tangential acceleration and deceleration are equal and it does not take into account the influence of normal acceleration limit (direction changes) in the printer speed. On the other hand, Habib and Khoda [29] also propose a simple trapezoidal speed profile model to estimate build time, nevertheless, the goal is not to make an accurate estimation of build time, but to use it to optimize the deposition direction. Moreover, some current computer applications, such as Pronterface [31], provide an analytical time estimation for FFF machines based on the printer characteristics, a trapezoidal speed profile and a cornering algorithm.

### 1.3. A Blended Solution

This work explains a new build time estimation model for FFF machines, which combines the analytical and experimental approaches. The proposed model takes into account the kinematics of the problem (as the analytical strategies) and defines it by fitting a low number of printing speed experimental observations. The solution is simpler than that of the analytical models and does not need to know the machine nominal parameters and how the printer is controlled. In contrast to parameter and experimental-approaches, the proposed solution requires only two parameters and few experimental tests to provide an accurate time prediction (estimation error below 8.5% in the printed examples). The experimental procedure requires a low-cost setup and can be easily accomplished with the explanation included in this paper. Moreover, unlike the experimental solutions, once the printing speed is approximated, the proposed method can be applied regardless of the chosen printing parameters.

Because of the printing path in FFF and tool path in a milling process are similar, our build time approach is obtained by modifying and extending the mechanistic model of Coelho et al. [32]. The proposed solution is based on a printing speed model, which not only considers the interpolation segment length, as the mechanistic model does, but also the path shape via the changes of direction. Path planning is key in the resulting build time of material extrusion processes [33]. Direction changes and small interpolation segments have a noteworthy impact on the actual time. Based on experimental observations, we show two different fitting strategies to determine the aforementioned printing speed model.

Some assumptions are considered. The method provides the time to print a part, without including the setup and heating times. Because of the fact that FFF motions are generally based on linear interpolation, the study is limited to this kind of interpolation scheme.

The remaining paper is organized as follows. Section 2 discusses the estimation procedure and Section 3 describes the experimental methods. Section 4 shows the approximation speed surfaces and validation examples. Finally, the main conclusions are drawn in Section 5.

## 2. Build Time Estimation Model

In this work, the way proposed to obtain the build time of actual parts is via the estimation of the actual printing speed. Through time measurements of known paths, we obtain information about how path definition (interpolation segment lengths and direction changes), machine characteristics, and its control influence the actual speed between consecutive interpolation points of the printing path. Known an approximation of the actual printing speed, time estimation is simple: reading the CNC printing code and using the speed approximation to estimate the real time of each path segment.

### 2.1. Average Printing Speed

**Figure 1:**
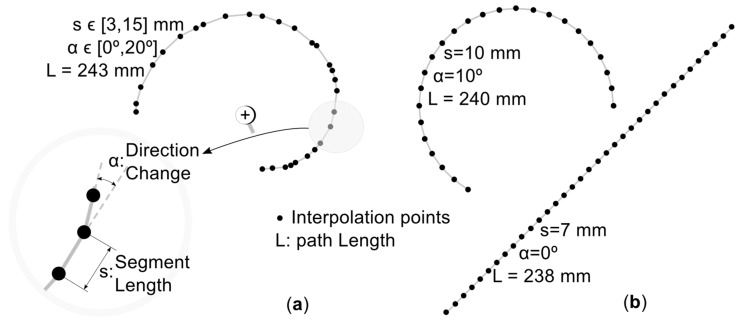
Examples: (**a**) Printing path with random segment lengths and direction changes, (**b**) printing paths for experimental speed estimation.

We assume hereafter that the main factors that influence the printing speed *f* are the interpolation segment length s and the direction change *α*. According to Figure 1a, *s* is the Euclidean distance between the two consecutive interpolation points. The direction change *α* is the angle between the direction of three consecutive interpolation points. To understand how *s* and *α* influence the printing speed, we measure the average speed along circular arcs (Figure 1b), which are built via repeating interpolation segments with the same length and direction change (a line with *α* = 0° corresponds to a circle of infinite radius). The speed measurement procedure is explained in Section 3.

**Figure 2:**
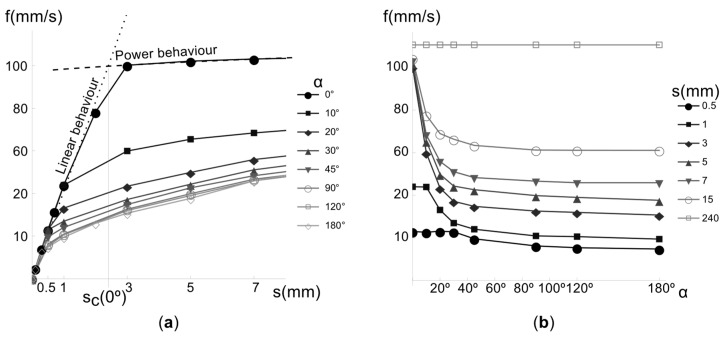
Example of experimental measurements of printing speed *f*. (**a**) Speed vs. *s* curves for different direction changes. (**b**) Speed vs. *α* for different segment lengths.

The speed-segment length relation evolves from linear to a power law (Figure 2a). The linear relation becomes smaller with increasing *α*, whereas the slope does not change. On the other hand, we have different power laws for each *α*; printing speed asymptotically decreases as *α* increases (Figure 2b).

Note that, we measure *f* from *s* = 0.1 mm to a maximum value *s*_Max_. We choose *s*_Max_ so that the circular path defined by interpolation segments with *s*_Max_ and *α* = 10° is the maximum circle within the printer bed.

Above *s*_Max_ it is more difficult to measure the printing speed. For this reason and based on the previous discussion, if *s* > *s*_Max_ we assume a power law. This power model is computed by interpolation of *f*(*α*, *s*_Max_) and *f*(0, *b*), where *b* is the printer bed diagonal length.

### 2.2. Printing Speed Surface

This section is devoted to determining an approximation surface that provides the printing speed for given values of *s* and *α*. There are different possibilities to define the printing speed surface *f*(*s*, *α*). The interpolation of the measurement points by means of a degree 1x1 polynomial spline patch provides a straightforward solution, but this approach requires many measurements to accurately predict time.

In order to reduce the number of needed measurements, we propose two alternatives:

- A linear-power (LP) surface that approximates the linear and power relation of *f* with respect to *s* as a function of the direction of change *α*.
- A spline of Coons patches (CP). Each Coons patch is defined by linear interpolation of four boundary curves.

To determine the isocurves *α* = constant on the LP surface, we approximate the segment length *s*_c_(*α*) where linear and power approximations intersect (Figure 2). The procedure consists of the next steps:

Step 1. Approximate the speed measurements at *α* = 0 and *s* < *s*_c_(*α* = 0) = *s*_c,0_ by a line *f*(0, *s*) = *L*(*s*) = *m·s + n*. The speed profiles, such as those portrayed in Figure 2a, show that if *s* < *s*_c_(*α*), then the linear relation *f*(0, *s*) does not change with *α*.
Step 2. Fit the speed measurements, within the interval *s*_c,0_ < *s* ≤ s_Max_, at *k* angles 0° ≤ *α_i_* ≤ 180°, *i* = [0, 1,…, k−1] by power curves $P_{i}\left(s\right)=a_{i}s^{b_{i}}$. Although *s*_c_ varies with *α*, the relation s_c,0_ > s_c_(*α*_i_) is satisfied, so that we always are in the region ruled by the power law, and fewer measurements are needed. Note that, by increasing *k* the approximation improves at the expense of accomplishing more measurements.
Step 3. Compute *s* at the intersection of *L*(*s*) and the power curves *P_i_*(*s*). Fitting the resulting data, for example by means of a degree-2 polynomial spline, we obtain the curve *s*_c_(*α*).
Step 4. Build the curves *f*(*α*, *s*_c_(*α*)) and *f*(*α*, *s*_Max_) and interpolate them using a power function $P\left(\alpha ,s\right)=a\left(\alpha ,s\right)s^{b\left(\alpha ,s\right)}$.
Step 5. Define the speed surface as a piecewise function:
  (1) $$f\left(\alpha ,s\right)\equiv \left\{L\left(s\right),\;s\leq s_{c}\left(\alpha \right);\;P\left(\alpha ,s\right),\;s_{c}\left(\alpha \right)<\;s\leq s_{\mathrm{Max}}\right\}$$

Regarding the CP, the idea is to define *f*(*α*, *s*) as a spline of two Coons patches. A Coons patch is a surface determined by its boundary curves, i.e., it is a way of filling the space between the curves.

The easiest Coons construction is a bilinear blend of two ruled surfaces and a bilinear interpolation surface [34]. Let *f*(*α*_0_, *s*), *f*(*α*_1_, *s*) and *f*(*α*, *s*_0_), *f*(*α*, *s*_1_) be the four parametric boundary curves, then it is easy to build a linear interpolation surface with each couple of curves:

(2) $$\begin{array}{c} r_{1}\left(\alpha ,s\right)=\left(1-\frac{\alpha -\alpha_{0}}{\alpha_{1}-\alpha_{0}}\right)f\left(\alpha_{0},s\right)+\frac{\alpha -\alpha_{0}}{\alpha_{1}-\alpha_{0}}f\left(\alpha_{1},s\right)\\ r_{2}\left(\alpha ,s\right)=\left(1-\frac{s-s_{0}}{s_{1}-s_{0}}\right)f\left(\alpha ,s_{0}\right)+\frac{s-s_{0}}{s_{1}-s_{0}}f\left(\alpha ,s_{1}\right) \end{array}$$

On the other hand, we can compute the bilinear interpolation of the four patch corners:

(3) $$r_{1,2}\left(\alpha ,s\right)=\left[1-\frac{\alpha -\alpha_{0}}{\alpha_{1}-\alpha_{0}},\frac{\alpha -\alpha_{0}}{\alpha_{1}-\alpha_{0}}\right]\left[\begin{array}{cc} f\left(\alpha_{0},s_{0}\right) & f\left(\alpha_{0},s_{1}\right)\\ f\left(\alpha_{1},s_{0}\right) & f\left(\alpha_{1},s_{1}\right) \end{array}\right]{\left[\left(1-\frac{s-s_{0}}{s_{1}-s_{0}}\right),\frac{s-s_{0}}{s_{1}-s_{0}}\right]}^{t}.$$

Finally, the Coons surface is:

(4) $$CP\left(\alpha ,s\right)=r_{1}\left(\alpha ,s\right)+r_{2}\left(\alpha ,s\right)-r_{1,2}\left(\alpha ,s\right).$$

A unique Coons patch with *f*(0, *s*), *f*(180, *s*) and *f*(*α*, 0), *f*(*α*, *s*_Max_) is unable to adequately reproduce the actual printing speed surface. To overcome this drawback, we define the printing speed *f*(*α*, *s*) as a spline of two Coons joined at the value of *s*_c,0_ defined in the same way as in the LP construction.

The following steps summarize the CP procedure:

Step 1. Measure the average speed at *α* = 0° and *α* = 180° for different segment lengths *s*, and at *s* = *s*_c,0_ and *s* = *s*_Max_ for several *α*.
Step 2. Fit the experimental data to obtain the boundary curves: *f*(0°, *s*), *f*(180°, s), *f*(*α*, *s*_c,0_), *f*(*α*, *s*_Max_). We approximate the experimental data by B-splines curves.
Step 3. Build two Coons patches: CP_A_ with *f*(0°, *s*), *f*(180°, s), *f*(*α*, 0), *f*(*α*, *s*_c,0_), and CP_B_ with *f*(0°, *s*), *f*(180°, s), *f*(*α*, *s*_c,0_), *f*(*α*, *s*_Max_).
Step 4. Compute the speed surface by the following spline function:

(5) $$f\left(\alpha ,s\right)\equiv \left\{CP_{A}\left(\alpha ,s\right),\;s\leq s_{c,0};\;CP_{B}\left(\alpha ,s\right),\;s_{c,0}\leq \;s\leq s_{\mathrm{Max}}\right\}$$

Note that, the approximation improves by increasing the number of Coons, but it requires increasing the number of measures.

Either for LP surface or the CP approximation, for *s* > *s*_Max_, the idea is to interpolate the curves *f*(*α*, *s*_Max_) and *f*(*α*, *b*) (that we assume equal to *f*(0°, *b*)) using a power function.

Section 4 portraits the resulting LP and CP approximation surfaces and shows the measurements used in both surfaces: 22 measures for LP and 25 for CP.

### 2.3. Build Time Estimation from G-Code

Once we have the printing speed surface, it is possible to determine the build time from the path G-code. Observe that, the time required for heating the filament and the bed, and the time needed by the hot-end to go home (setup time) are not considered.

The computation process consists of:

- Read the ISO code and obtain the printing path in each layer.
- For each interpolation segment *j*, determine the programmed printing speed *f*_p*j*_, its length *s_j_*, and the direction change *α_j_* of the segment respect to the previous one.
- Choose the printing speed surface according to the machine and estimate the actual printing speed *f*(*α_j_*, *s_j_*).
  We take the programmed speed for *z* movements, as well as for the hot-end reposition when motors in *x-y-z* axis work at the same time because only the printing speed of *x-y* motors is measured.
- Finally, if the path has *l* segments, the estimated build time *t* is:

(6) $$t=\overset{l}{\underset{j=1}{\sum }}\frac{s_{j}}{f_{a}},\;\;\;\;\;f_{a}=\left\{f\left(\alpha_{j},s_{j}\right),\;if\;f\left(\alpha_{j},s_{j}\right)<f_{pj};\;f_{pj},\;\mathrm{Otherwise}\right\}.$$

In order to accomplish the above procedure, we write a Mathematica^®^ function. This function reads a G-code file, distinguishes each layer and detects the programmed printing speed along the layer paths. After that, it obtains the coordinates of the interpolation points and computes segments lengths *s* and, using the dot product, angles *α*. Finally, the developed function uses Equation (6) to estimate the actual build time.

## 3. Materials and Methods

We use the previously described estimation method (Section 2) to obtain the build time in three low-cost FFF machines: BQ Hephestos^®^, Witbox^®^, and Airwolf 3D HD^®^ printers. 2D dimensional paths with random lengths and random direction changes, hereafter referred to as “random paths,” and actual 3D printed parts are designed to validate our time estimation procedure.

### 3.1. Printers

Table 1 shows the main characteristics of the tested 3D printers. Extrusion and movements along *x*, *y*, and *z* axes are powered by standard stepper motors. We use the same travel speed for all printers: 120 mm/s.

### 3.2. Speed Measurement Procedure

In order to measure the speed at a specific *s* and *α*, we drive the printer hot-end through interpolation segments of length *s* and direction change *α* (circular paths with total length *L* ≈ 240 mm, Figure 1b), measure the build time *t*, and finally compute the average speed as the ratio of *L* to *t*.

A chronometer can lead to inaccurate time measurements, and even more for short paths. Thus, we decided to implement a measurement procedure based on producing a sound at the ends of the printing path, which leads to a clear identification of the build time. For printers with a Marlin firmware, such as those studied in this work, this means to add the following line before the first printing path position and after the last one:

“M300 P200 S440; play a 440 Hz tone during 200 ms.”

A speaker to run the previous instruction is required. For a printer without a speaker, it is easy to connect a buzzer to an empty port of its electronic card and use the previous command. Witbox machine has a speaker, meanwhile, the Airwolf and the Hephestos machines need a buzzer.

Sound is recorded by a microphone connected to a PC, which allows distinguishing the time between the start and end. Each test was run three times. To determine the speed measurement uncertainty *u*(*f*), we apply the combined standard uncertainty [35] to the equation *f* = *L*/*t*. The maximum *u*(*f*) obtained was lower than 0.2 mm/s for the three studied machines. 

### 3.3. Experimental Tests

In addition to the speed observations for building the approximation surfaces discussed in Section 3.2, we design two types of validation tests: random paths and printing examples.

All tests were conducted in the Pronterface application. Pronterface, similar to other 3D printing applications, provides an analytical print time estimation based on the planner functions used by the printer firmware to define the printer kinematics. These functions are a model of the speed, which by default is a trapezoidal profile, and a cornering algorithm that deals with the direction changes, and it is usually based on a limit jerk equation. The performance of analytical models depends on the nominal parameters of motors and control, whose values can differ from the real ones, and an adequate definition of factors such as the jerk limit.

It is interesting to compare the proposed model with a 3D printing software estimation, as many researchers [36,37,38,39] trust on those predictions to conduct costs and process optimization studies.

#### 3.3.1. Random Path Tests

A set of random paths are tested to assess the performance of the proposed estimation procedure in specific *s*-α regions. Six paths, with a similar total length of 2400 mm, composed of segments with random lengths and directions (random paths) are conducted at travel speed without extrusion in each printer. A Mathematica function is implemented to provide the random paths and to write the needed G-code file. This function defines *x*-*y* positions and the reference printing speed (travel speed).

#### 3.3.2. Printing Examples

We design three 12 mm high prisms with simple bases (triangle, pentagon, and star) at two scales (1:1, 2:1), and print them using two printer configurations. The well-known software Ultimaker CURA^®^ (Free and open source LGPLv3 application developed and maintained by David Braam for Ultimaker, a 3D printer manufacturer based in Utrecht, Netherlands) provides the G-code. This software provides a similar time estimation to that of Pronterface, but it does not allow estimating times from G-codes obtained outside the software, such as for the random paths. For this reason, we compare our results to Pronterface predictions instead of Ultimaker CURA estimations.

Table 2 shows the factors and levels considered, and Table 3 summarizes the 12 tests performed corresponding to all possible combinations for the considered factors and levels, and the actual printing time measures. Note that, geometry, size, and printing parameters modify the *s*-α values and therefore, the resulting build time.

## 4. Results and Discussion

The present section is organized as follows: Section 4.1 shows the experimental results required to build the two proposed approximation surfaces described in Section 2.2 (LP and CP surfaces), as well as the resulting surface models. Section 4.2 presents several tests for assessing the accuracy of the proposed models. This validation procedure consists of carrying out several paths where the printing time is recorded and compared to that provided by each approximation model. In Section 4.2.1, this procedure is applied to random paths (without extrusion of printing material), with the target of facilitating the variation in direction and segment length of a trajectory and analyzing their influence on printing time estimation. In Section 4.2.2., the validation procedure is also applied to several printed parts with different geometries in order to find out if the results provided by the approximation surfaces are also accurate for actual examples. A discussion regarding a comparison between the proposed methods, as well as between them and some usual methods for estimating the printing time, such as the Pronterface and theoretical estimations, is included at the end of the present section.

### 4.1. Printing Speed Measurements

**Figure 3:**
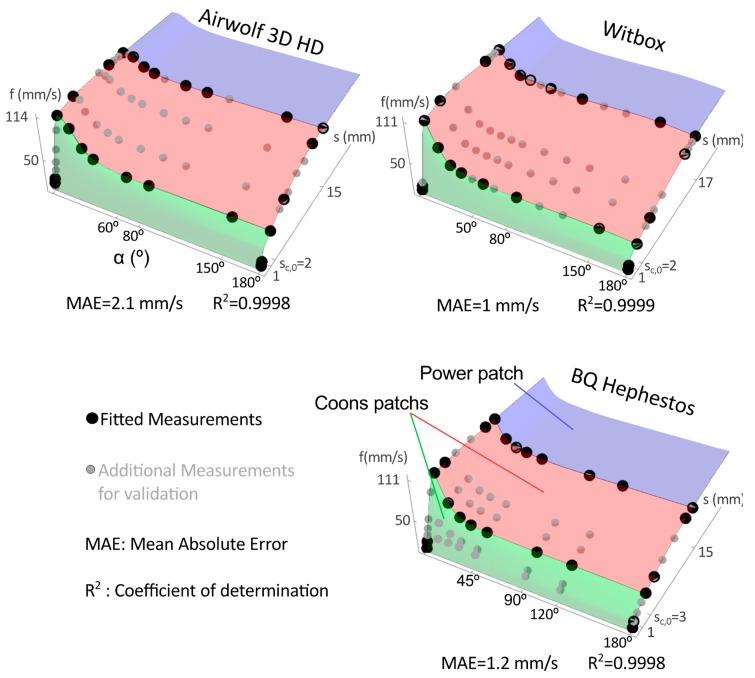
Coons patches (CP) printing speed surfaces for the tested printers (different colors are used to identify each patch of the CP spline surface). Dots represent the experimental measurements, CP surfaces fit black dots, and additional measurements (gray dots) are represented to visualize the goodness of the approximation.

**Figure 4:**
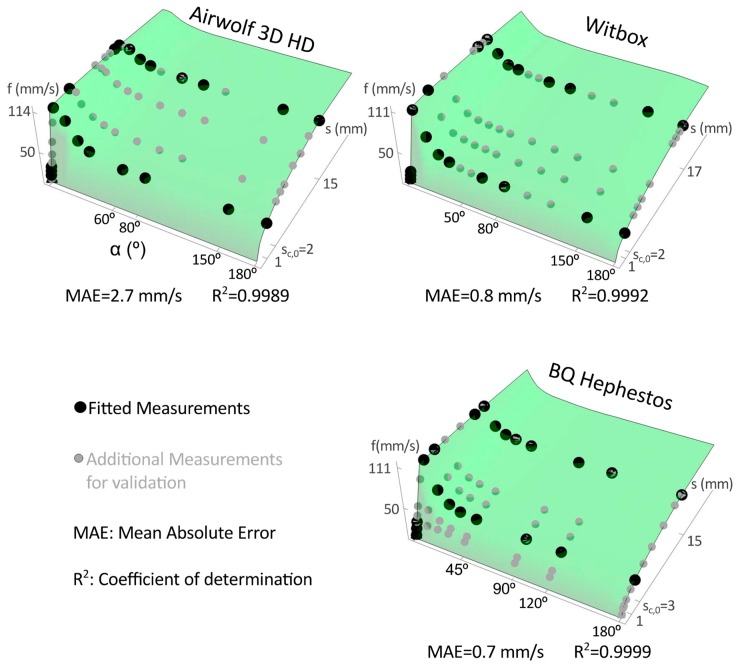
Linear-power (LP) printing speed surfaces for the tested printers. Dots represent the experimental measurements, LP surfaces fit black dots, and gray dots are additional measurements to visualize the goodness of the approximation.

Figure 3 and Figure 4 show the printing speed measures and the proposed speed estimation models: LP and CP surfaces obtained. Black dots depict the measures used for the approximations, and grey dots represent additional measures used to verify the goodness of the approximation.

The mean absolute error (MAE) of all measurements and the determination coefficient R^2^ of the approximations are included in Figure 3 and Figure 4. The goodness of the approximations is high for all printers since R^2^ is close to 1 and MAE value is low.

Note that, we choose a reduced number of measures to be fitted (similar for LP and CP approximations). The idea is to take measurements close to *s* = 0, *s*_c_ and *s*_Max_ at α = 0°, 180°, and at *s*_c,0_ and *s*_Max_ for different angles (black dots in Figure 3 and Figure 4). The linear region, for the tested printers, is always obtained for segment lengths lower than 1 mm so that, to capture this behavior we take measurements every 0.1–0.2 mm from *s* = 0. On the other hand, the power region is wider than the linear one and, therefore, we use steps of 2–5 mm from *s*_c_ and *s*_Max_. Regarding the data at *s*_c_ and *s*_Max_ for different angles, it is better to take more measurements between α = 0° and α = 60° because the greatest variations are registered within this range. Considering the previous suggestions, we obtain similar surfaces when fitting different experimental data.

LP and CP surfaces are similar and evolve as it is expected considering a trapezoidal speed profile and the machine acceleration limits. With respect to *s*, the surfaces evolve from linear to power, and that is explained because in each interpolation segment the printer accelerates to reference speed and decelerates up to the segment end (trapezoidal speed profile). On the other hand, the speed decreases with α (mainly between 0° and 60°). It stands to reason that the curvature and printing speed have a quadratic relation, which agrees with the experimental data and with the LP and CP surfaces obtained.

Machine characteristics influence the resulting speed surface. Although the surfaces have similar shapes, the Airwolf surfaces (LP and CP) provide the highest speed values and the Hephestos the lowest values in the considered *s*-α domain. In the Airwolf machine, the linear region grows steeper than in the other printers whereas the power region grows smoother. It stands to reason that the actual average speed depends on the printer acceleration limits (see Table 1) and this explains the above results.

### 4.2. Validation Tests

#### 4.2.1. Random Paths

**Figure 5:**
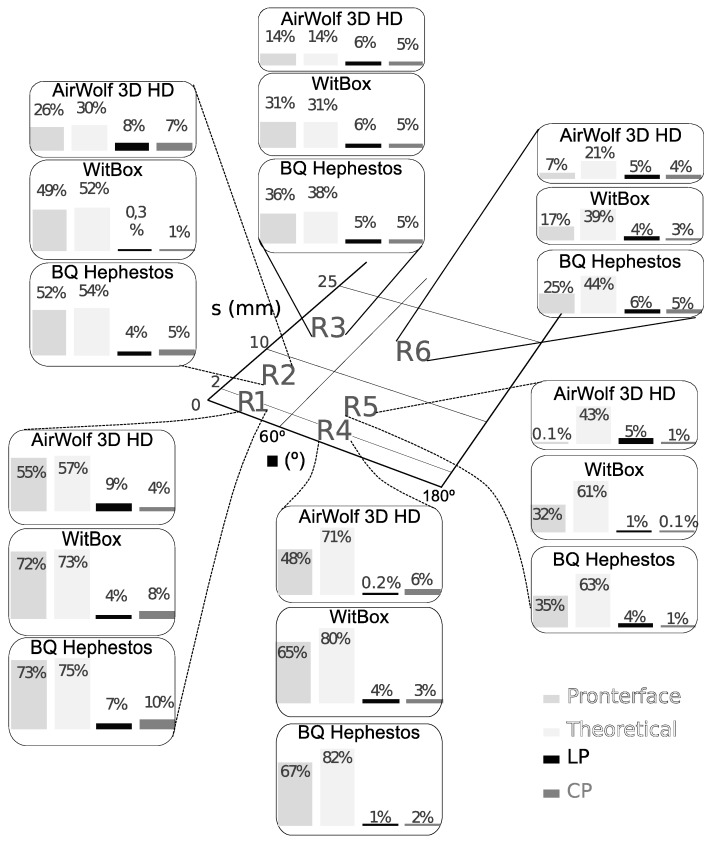
Estimation relative error for six random paths at different regions of the *s*-*α* domain.

Six random paths (Section 3.3.1) were printed using the Pronterface application. Figure 5 shows the resulting actual printing time, the theoretical estimation (sum of ratios of segment length to programmed speed), the Pronterface prediction and the print time estimation provided by the LP and CP surfaces in the tested printers.

Comparing the six examples, the proposed approximations provide the most accurate estimations. In each printer, LP and CP average errors are similar and always lower than 5.5%. This value improves Pronterface and theoretical average errors, which are up to 48% and 59% respectively. The dispersion observed in the error values is a consequence of how close the approximations are to the actual printing speed surfaces at each region *s*-α.

Speed mainly changes within the linear region, in the transition from linear to power and because of direction changes up to 60° (see Figure 3 and Figure 4). Thus, Pronterface, LP, and CP errors have maximum values at R1 and R2. Regarding the theoretical error, it does not consider *s* and α variations, which leads to a maximum error in R4. On the other hand, theoretical and Pronterface predictions are quite similar in regions R1 to R4, but Pronterface estimation improves when α increases (regions R5 to R6) because it considers the cornering algorithm used by the printer control.

Finally, comparing the LP and CP approximations in the printers, the Airwolf speed surface has a MAE greater than those obtained for the Witbox and Hephestos printers (see Figure 3 and Figure 4), and this leads to the differences observed in the time estimation error values.

#### 4.2.2. Printing Examples

To assess the performance of the proposed estimations in real parts, 12 prisms are printed (see Table 3). Examples in the previous Section 4.2.1 point out that estimation error depends on the *s*-α region, but for actual printed parts, *s* and α values are not concentrated in a specific region and that can reduce the prediction errors. Another fact that contributes to differentiate random paths and printing examples is the programmed printing speed. While for random paths, the programmed speed is 120 mm/s, which is always greater than the experimental maximum speed measured, in the printing examples the programmed speed changes along the printing path and can be beneath the actual maximum speed surface.

**Figure 6:**
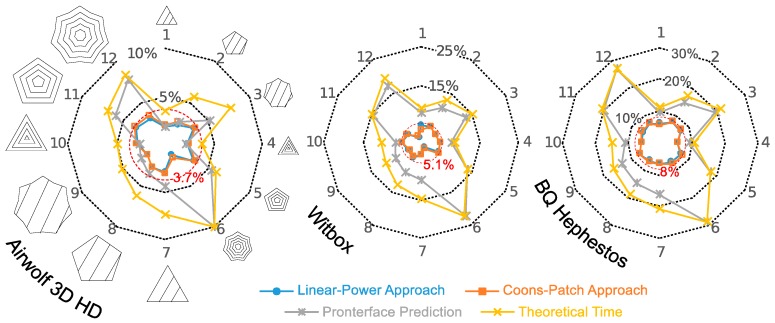
Relative error of the proposed method, theoretical estimation and Pronterface prediction for the twelve printed samples presented in Table 3.

Figure 6 portrays the relative errors corresponding to the theoretical estimation (considering the programmed printing speeds), the Pronterface prediction and the estimations provided via our LP and CP surfaces.

According to Figure 6, it is easy to observe the improvement in printing time prediction provided by the proposed approaches in comparison with theoretical and Pronterface estimations. For the 12 printed examples, while theoretical and Pronterface approaches show dispersed error values with maximum values in samples 6 and 12, and minimum values in samples 1 and 4, LP and CP solutions show similar relative errors that never exceed 8.5%.

Comparing the printers, the Hephestos shows the worst results followed by the Witbox, and the Airwolf shows the minimum error. With respect to LP and CP estimations, this result differs from those obtained for the random paths, but it makes sense considering that, for the printing examples, the programmed speed can have a value below the maximum actual speed surface. In this case, the estimations consider a speed equal to the programmed speed (see Equation (6) for LP and CP solutions). Estimation errors decrease with the difference between programmed and average actual speed. Printer acceleration determines that difference, in a manner that, the fastest printers show the lowest estimation errors in the printing examples.

## 5. Conclusions

The printing speed predictions showed in this paper lead to accurate build time estimations (maximum relative error of 8.5 % in the printed examples). The experimental methodology devised to build a printing speed surface can be straightforwardly applied to any FFF or similar machines, by measurement printing times for different segment lengths and direction changes along linear and circular paths. The proposed fitting procedures, LP and CP approaches, provide good mean printing speed approximations (mean absolute error lower than 2.7 mm/s) even for a reduced number of experimental data (22 measurements for LP and 25 measurements for CP).

The estimation procedure requires to read the G-code that defines the printing path. For each interpolation segment, the method compares and chooses the lowest speed between the programmed and predicted one, and computes the required time to travel the segment length at that speed.

It is noteworthy that the proposed method was successfully applied to three low-cost FFF printers. In the experimental tests accomplished (six random paths and twelve printed prisms), LP and CP estimations provide the minimum errors. In many cases, these errors are well below those provided by theoretical and Pronterface (analytical) estimations. Moreover, for all tested printers, while the theoretical and Pronterface estimation errors show high dispersion, CP and LP errors hardly change. Hence, the infill pattern and the component shape and size do not modify the accuracy of the proposed approach.

LP and CP surfaces are defined for a specific maintenance state of the printers, and it stands to reason that wears or maintenance problems could increase time estimation error. Further effort is required to study this fact, which could help to find out when to start maintenance tasks in a FFF machine.

## Figures and Tables

**Table 1:** Main technical characteristics of tested 3D printers.

	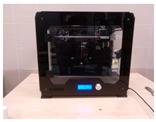 Witbox	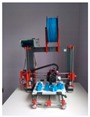 BQ Hephestos	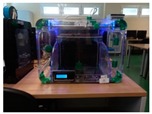 Airwolf 3D HD
Manufacturer	Mundo Reader, S.L. (Madrid, Spain)	Mundo Reader, S.L. Madrid (Madrid, Spain)	Wolf & Associates Inc. (Costa Mesa, CA, USA)
Build volume (*x*, *y*, *z*) (mm)	297 × 210 × 200	215 × 210 × 180	300 × 200 × 300
Minimum layer thickness (μm)	50	60	60
Filament diameter (mm)	1.75	1.75	3
Nozzle diameter (mm)	0.4	0.4	0.5
Max. recommended print speed (mm/s)	80	60	100
Maximum acceleration (mm/s^2^)	1000	1100	2500
Circuit board	Mega 2560	BQ Zum Mega 3D	RAMBo
Hot-end movement	*x*,*y*	*x*,*z*	*x*,*y*

**Table 2:** Factors (parameters) and levels (factor values) to define the printing examples.

	Levels		
**Factors**	−1	0	1
Shape	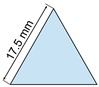	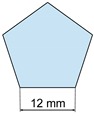	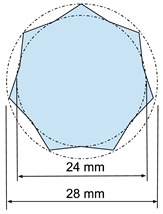
Scale	1:1	-	2:1
Printer configuration	Pattern: Zig-zag	-	Pattern: Concentric
	Top-Bottom thickness: 0 mm		Top-Bottom thickness: 0.2 mm
**Default configuration for the tested printers:**			
0.2 mm Layer Height Material: PLA Brim: 2 mm Infill density 15% Wall thickness 0.5 mm No support		Reference Speeds (mm/s) ○ Print: 40 ○ Infill: 80 ○ Wall: 20 ○ Top-Bottom: 15 ○ Travel: 120	

**Table 3:** Actual printing time measured for the accomplished tests of Table 2.

Test	Factors			Real Printing Time (s)		
	Shape	Scale	Configuration	Air-wolf	Witbox	Hephestos
1	−1	−1	−1	324	344	353
2	0	−1	−1	339	367	386
3	1	−1	−1	469	509	551
4	−1	−1	1	346	364	372
5	0	−1	1	389	424	447
6	1	−1	1	608	702	769
7	−1	1	−1	932	1014	1094
8	0	1	−1	1318	1417	1524
9	1	1	−1	2351	2498	2671
10	−1	1	1	1035	1103	1155
11	0	1	1	1560	1709	1836
12	1	1	1	2936	3346	3693
